# Supplementary material for: Polymorphisms in Pfkelch13 domains before and after the introduction of artemisinin-based combination therapy in Southwest Nigeria
Source: PLoS One. 2025 Mar 31;20(3):e0316479. doi: 10.1371/journal.pone.0316479 (PMC11957316; doi:10.1371/journal.pone.0316479)

Samples: 12665
Bases: 1066
Average spacing: 12

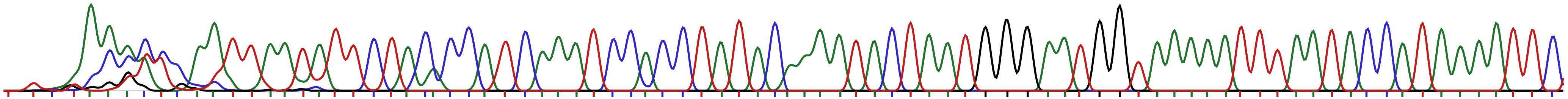

T T C T A A A C T C A A T T A A T A T T C T A C A C C A T C A A A T C C A C C T A T A C A A A A T A C T A A T G G G A A T G G T A A A A A T T T A A T A C C A T A A A A T T C

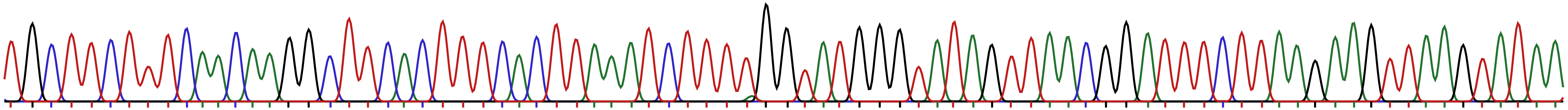

T G C T T C T T T C A A C A A G G C T T C A C T T T C A C T T A A A T C T T T T G G T A T G G G T A T A G T T A A C G G A T T T C T T A A G A A G T T A A G T A T A A

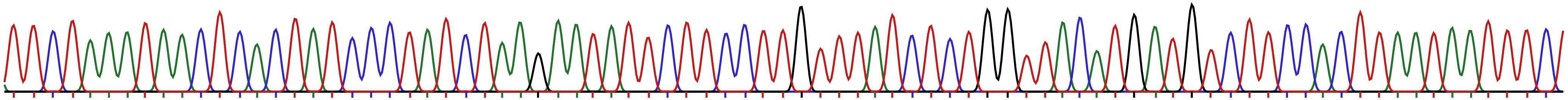

T T C T A A A T A A C T C A C T A T C C C T A T C T A A G A A T A T T C T T C C T T G T T T A T C T C T G G T T A C A T G A T G T C T T C C A C T T A A T A A T T T C

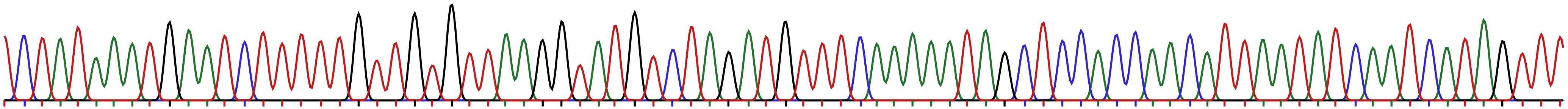

T C T A T A A A T G A A T C T T T T T G T T G T G T T A A G G T A T G T C T A G A T G T T T C A A A A A T A G C T C C A C C A A C A T T A A T A T C A A T C A T A G T T T

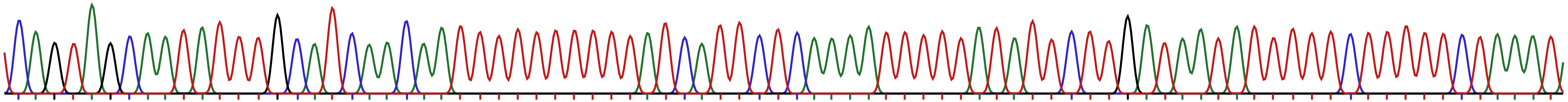

C A G T A G C A A T A T T T G C A T C A A C A A T T T T T T T T T A T C A T T C T C A A A A T T T T T A T A T T C T T G A T A A T A T T T T T C T T T T T C T A A A T

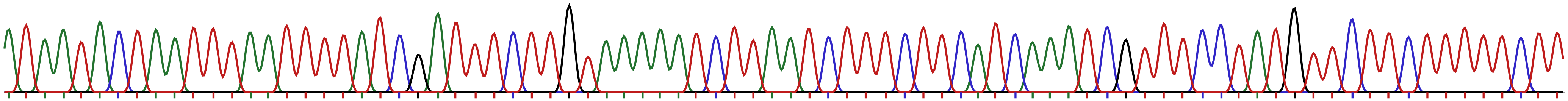

A T A A T A C T A A T T T A A T T T T A T C G A T T T C T T G T A A A A A T C T T A A T C T T T C T T C A T C A A A T C G T T T C C T A T G T T C T T C T T T T T C T T

Samples: 12665
Bases: 1066
Average spacing: 12

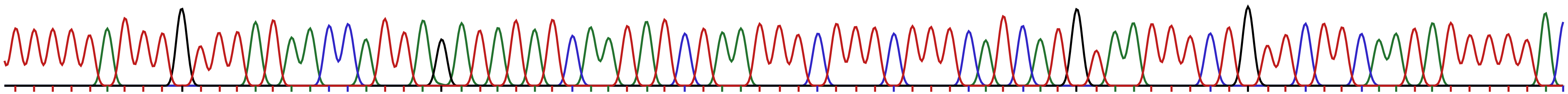

510 520 530 540 550 560 570 580 590

T T T T T A T T T G T T T A T A A C C A T T A G A T A T A T C A A T A T C T A A T T T C T T T C T T T C A T C A T G T A A T T T C T G T T C T T C A A T A T T T T T A C

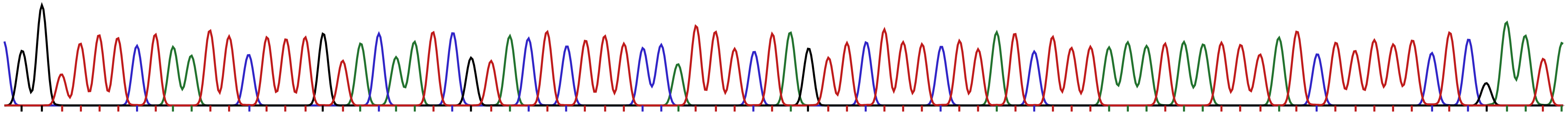

600 610 620 630 640 650 660 670

G G T T T T C T A A T T C T T T G T A C A A T C G T A C T C T T T C C A T T T C T A G T T C T T T C T T A T C T T T A A A T A A T T T A T C T T T T T C T C G A A T A

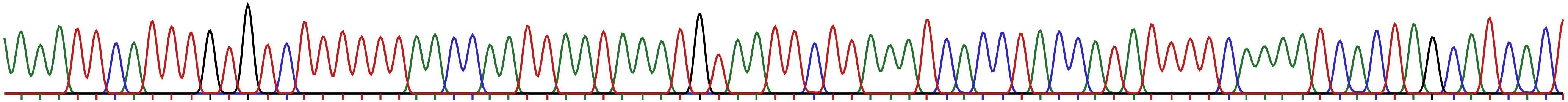

680 690 700 710 720 730 740 750

A A A T T C A T T T G T G T C T T T T T T A A C C A A T T A A T A A A T G T A A T T C T T A A A T C A C C T A C C A T A T T T T C A A A A T C A C T A G C A T C A C T

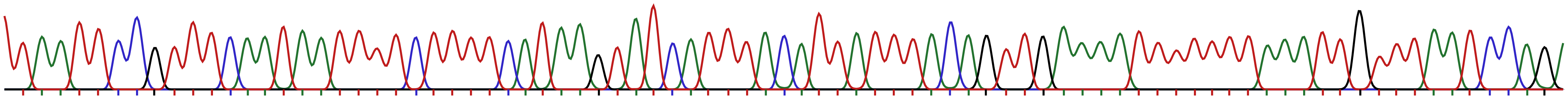

760 770 780 790 800 810 820 830

T A A T T C C G T T T C A A T A A T T T T C T T T T C A T A A G T A T C A T T T A C A T T A T T T A C A G T T G A A A A T T T T T T T A A A T T G T T T A A T C C A G

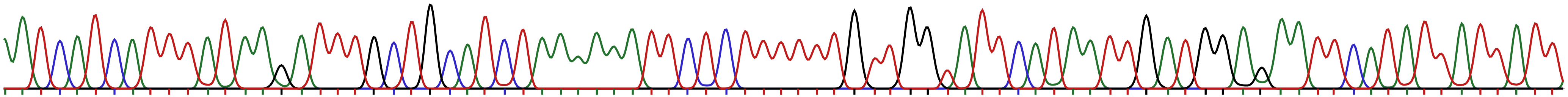

840 850 860 870 880 890 900 910 920

A A T C A T C A T T T A T A A G A T T T G C T G C A T C T A A A A A A T T C T C T T T T T T G T T G G T A T T C A T A A T T G A T G G A G A A T T C A T A T T A T T A T T

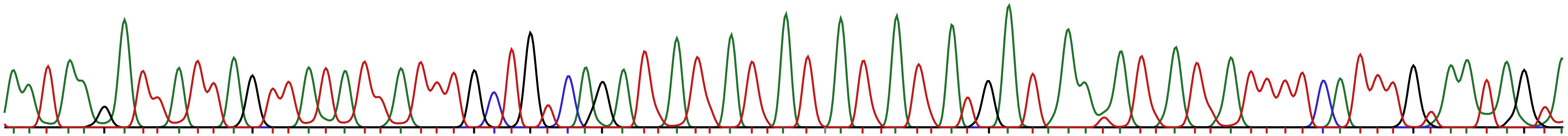

930 940 950 960 970 980 990 1000

A A T A A G A T T A T T A G T T A T A T T A T T T G C T G T C A G A T T A T T A T T A T - A T T A T T A T G A T A A A T A T T A T T A T T T T C A T T T G T A A T A G T A

Samples:	12665	
Bases:	1066	
Average spacing:	12	

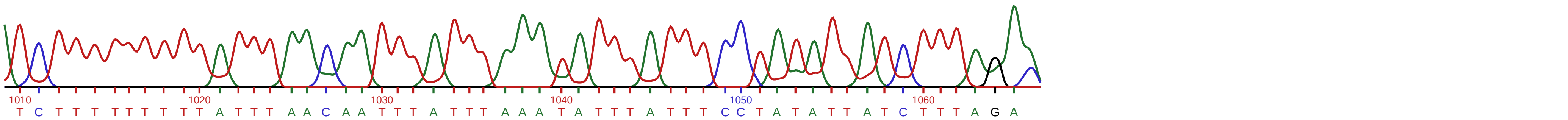

Supplement: Supporting information 2 — (ZIP) [file pone.0316479.s002.zip › 075KN2R_PREMIX_Plate_KELCH2_G10.pdf]
